# Safety and Effectiveness of Blonanserin in Chinese Patients with Schizophrenia: An Interim Analysis of a 12-Week Open-Label Prospective Multi-Center Post-marketing Surveillance

**DOI:** 10.3389/fpsyt.2022.935769

**Published:** 2022-08-18

**Authors:** Haishan Wu, Xijin Wang, Xuejun Liu, Hong Sang, Qijing Bo, Xiaodong Yang, Zhiyuan Xun, Keqing Li, Ruiling Zhang, Meijuan Sun, Duanfang Cai, Huaili Deng, Guijun Zhao, Juhong Li, Xianglai Liu, Guilai Zhan, Jindong Chen

**Affiliations:** ^1^National Clinical Research Center for Mental Disorders, Department of Psychiatry, China National Technology Institute on Mental Disorders, The Second Xiangya Hospital of Central South University, Changsha, China; ^2^The First Psychiatric Hospital of Harbin, Harbin, China; ^3^Brain Hospital of Hunan Province, Changsha, China; ^4^Changchun Sixth Hospital, Changchun, China; ^5^Beijing Anding Hospital, Capital Medical University, Beijing, China; ^6^Shandong Mental Health Center, Jinan, China; ^7^Tianjin Anding Hospital, Tianjin, China; ^8^Hebei Provincial Mental Health Center, Baoding, China; ^9^Henan Mental Hospital, Xinxiang, China; ^10^Daqing Third Hospital, Daqing, China; ^11^The Fifth People's Hospital of Zigong, Zigong, China; ^12^Psychiatric Hospital of Taiyuan, Taiyuan, China; ^13^Guangyuan Mental Health Center, Guangyuan, China; ^14^The Fourth People's Hospital of Chengdu, Chengdu, China; ^15^Hainan Provincial Anning Hospital, Haikou, China; ^16^Xuhui Mental Health Center, Shanghai, China

**Keywords:** blonanserin, safety, effectiveness, post-marketing surveillance, schizophrenia

## Abstract

Schizophrenia is an unexplained, complex and serious mental illness. Blonanserin (BNS) is a new antipsychotic drug widely used in the treatment of schizophrenia. However, large-scale clinical studies have not been conducted in China. A multi-center, prospective, open-label, 12-week surveillance was carried out to evaluate the safety and effectiveness of BNS in patients with schizophrenia in China. Safety assessments included adverse drug reactions (ADRs), extrapyramidal symptoms (EPS), akathisia, concomitant medications for EPS by the end of treatment, and the changes in body weight from baseline by the end of treatment. The effectiveness was evaluated by the Brief Psychiatric Rating Scale (BPRS). From September 2018 to May 2020, of the 1,060 patients enrolled, 1,018 were included in the full analysis set (FAS) and safety set (SS), respectively. ADRs were developed in 205 patients among the included, the incidence being 20.1%. ADRs of EPS occurred in 169 patients, the incidence being 16.6%, ADRs of akathisia occurred in 90 patients, the incidence being 8.8%; concomitant therapeutic and prophylactic agents for EPS accounts for 19.2%; 4.0% of patients had a ≥7% increase in body weight from baseline at 12 weeks after initiating treatment. Using the last-observation-carried-forward (LOCF) method, the changes in total BPRS scores were −11.2 ± 10.17 (*N* = 1,018), −16.8 ± 12.69 (*N* = 1,018) and −20.6 ± 13.99 (*N* = 1,018) after 2/4, 6/8, or 12 weeks, respectively. 53.5% (545/1,018) patients showed response to blonanserin treatment in week 12. The post-marketing surveillance results of BNS demonstrates safety profile and effectiveness of the drug.

## Introduction

As an incurable disease, schizophrenia is a unexplained, complex and serious mental illness, which affects about 21 million people worldwide. It is mainly manifested by two different types of symptoms, positive symptoms (such as hallucinations, delusions, etc.) and negative symptoms (such as apathy, lack of will, etc.) (1).

Unfortunately, at least one-third of patients with schizophrenia show no response to existing drugs according to the survey (2). Besides, most patients discontinue treatment due to serious adverse drug reactions (ADRs), such as weight gain, movement disorders, etc. There is an urgent need to find new drugs to increase patients' drug sensitivity, reducing the adverse effects. Blonanserin (BNS; LONASEN^®^) is a new type of atypical antipsychotic, synthesized in the early 1980s.

It is a dopamine receptor and serotonin receptor antagonist. *In vitro* receptor binding test, BNS has affinity for dopamine D2 receptor subtypes (D2, D3) and 5-HT2A receptor, as well as its main metabolite N-deethyl body (3). The binding of these receptors is related to the drug's therapeutic effect on schizophrenia. Furthermore, BNS and N-deethyl body has low affinity for adrenaline α1, histamine H1, and muscarinic M1 receptors, which is related to the possible complications during the treatment of the drug. The clinical trials of BNS conducted in Japan, South Korea, and Europe consistently show that its efficacy is comparable with haloperidol and other second-generation antipsychotic drugs such as risperidone (4–7). The incidence of side effects is lower, such as weight gain and prolactin elevation. Additionally, the results of pharmacological experiments, pharmacokinetic experiments and toxicity experiments also show that it has good safety profile (8).

A number of clinical trials have been carried out to verify the efficacy and safety of BNS in Chinese patients with schizophrenia. From February 2012 to February 2013, the results of a randomized, double-blind, double-dummy, parallel-controlled multi-center phase III clinical trial with risperidone as the control drug showed that the efficacy of BNS for schizophrenia is comparable to that of risperidone (7). In the safety test of the drug, clinical trials conducted in China were consistent with clinical trials in Europe and America (5), Japan (5, 8), and South Korea (6, 9). No new ADRs or trends were reported. The results of phase III clinical trials in China also showed that BNS has a lower incidence of causing blood prolactin elevation, weight gain, and heart-related abnormalities, compared with risperidone (10). Though there are some clinical trial data available in China, the sample size is still small. According to the Chinese government's “guideline for post-marketing surveillance of medicines (draft)”, post-marketing surveillance (PMS) is also required for BNS. This study aims to carry out a surveillance of the actual use of BNS in patients with schizophrenia in normal clinical practice and the safety profile of BNS in the Chinese population and evaluate effectiveness of BNS alone or in combination with other antipsychotic drugs.

## Patients and Methods

### Methods

From September 2018 to May 2020, 1,060 patients with schizophrenia were recruited from 16 sites across China.

All patients taking BNS within a period of time are enrolled for followed-up study. The full analysis set (FAS) includes all patients who at least received one blonanserin treatment. Efficacy analyses were performed based on the FAS. All safety analyses were performed based on the safety set (SS), which included all patients who received at least one blonanserin treatment. Electronic data capture (EDC) system is used to collect the diagnosis and treatment information from the patients (including but not limited to laboratory examinations, electrocardiograms, etc.) to obtain drug use, ADRs and other related information. The study was approved by the ethics committee of leading site. All patients provided written informed consent after a complete explanation unless informed consent approved by the site where the patients were enrolled is waived.

### Safety Evaluation

Adverse events (AEs) were coded according to the ICH International Dictionary of Medical Terms (MEDDRA 21.0, Medical Dictionary for Regulatory Activities). AEs and ADRs during the treatment period were reported by the participating physicians. ADRs were defined as AEs whose causality to BNS could not be ruled out, determined by the participating physicians.

### Effectiveness Evaluation

The severity of schizophrenia was evaluated using the Brief Psychiatric Rating Scale (BPRS) (11, 12) at baseline, 2/4, 6/8, and 12 weeks. The endpoint was the mean change in BPRS total score from baseline to the end of treatment (day 1 as baseline). The BPRS scale is an 18-item, 7-point rating system with a score for each item in the range 1–7, and a total score in the range 18–126. The BPRS scale can be divided into five factors (11): anxiety-depression (anxiety, guilt, depression, and somatic concern); anergia (emotional withdrawal, motor retardation, blunted affect, and disorientation); thought disturbance (conceptual disorganization, grandiosity, hallucination, and unusual thought content); activation (tension, mannerisms and posturing, and excitement); hostility-suspiciousness (hostility, suspiciousness, and uncooperativeness). The 5-factor model scores were collected. Rate of response (defined as improvement ≥ 40% at week 12 from baseline in BPRS total scores) was also included to evaluate the effectiveness (13).

### Statistical Analysis

SAS 9.4 software was used for analysis. Categorical variables were represented as *n* (%) and continuous variables were summarized as mean ± standard deviation (SD). Efficacy endpoint data (i.e., BPRS scores) were analyzed using the last-observation-carried-forward (LOCF) approach for the missing values. Paired t-test, analysis of variance (ANOVA) were used to compare continuous variables while χ^2^ analysis was used for categorical variables. Statistical significance was defined as *P* < 0.05.

## Results

### Baseline Demographics and Clinical Characteristics

Demographic data analysis showed that 39.0% of the patients were male and 61.0% female, with an average age of 32.9 ± 13.26 years old. 7.7% of the patients were younger than 18 years old, 67.2% were between 18 and 40 years old, and 25.1% were over 40 years old. 98.8% were Han nationality and 1.2% were others; The average height is 169.0 ± 7.43 cm. The average body weight was 65.8 ± 14.16 kg (Table 1).

**Table 1:** Demographics of the patients with schizophrenia.

**Background factor**	**Category**	**BNS group (*n* = 1,018)**
Sex	Male	397 (39.0)
	Female	621 (61.0)
Age	Mean ± SD	32.9 ± 13.26
	<18 years	78 (7.7)
	≥18 and ≤ 40 years	684 (67.2)
	>40 years	256 (25.1)
Height (cm)	Mean ± SD	169.0 ± 7.43
Weight (kg)	Mean ± SD	65.80 ± 14.163
Duration of illness (month)	Mean ± SD	63.80 ± 102.311
Baseline BPRS score	Mean ± SD	48.7 ± 13.48
Baseline diseases	Yes	107 (10.5)
Baseline medicine	Yes	505 (49.6)

*BPRS, Brief Psychiatric Rating Scale*.

### Dosage

For the 1,018 patients, the mean daily BNS dose during the treatment was 11.3 ± 4.18 mg. The mean model dose was 11.8 ± 4.79 mg/d over the treatment period for all patients.

### Safety of BNS

**Figure 1:**
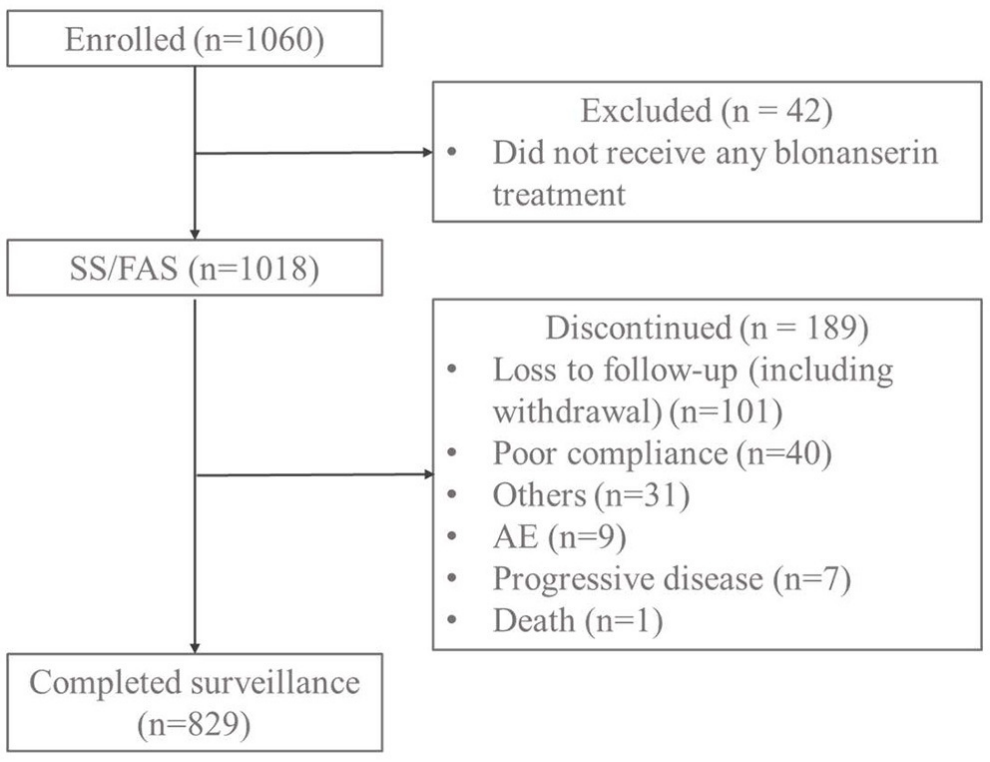
Patient disposition.

Forty-two (4.0%) were excluded among the 1,060 patients enrolled, because they were found not receiving any blonanserin treatment. Eight hundred and twenty-nine (78.2%) continue administration up to 12 weeks. A total of 189 patients discontinued administration. Loss to follow-up (including withdrawal) is the most common reason (Figure 1). Two hundred and five patients developed ADRs among the 1,018 patients included, the incidence being 20.1%. ADRs of EPS occurred in 169 patients, the incidence being 16.6%. ADRs of akathisia occurred in 90 patients, the incidence being 8.8%. Subgroup analysis showed that 205 mild ADRs occurred in 142 patients (13.9%). Eighty-six moderate ADRs occurred in 57 patients (5.6%). Seven severe ADRs occurred in 6 patients (0.6%) (Table 2).

**Table 2:** ADRs involve systematic and clinical manifestations.

**ADRs**	**Mild**		**Moderate**		**Severe**	
	**Number of patients,** ***n* (%)**	**Number** **of cases**	**Number of patients,** ***n* (%)**	**Number** **of cases**	**Number of patients,** ***n* (%)**	**Number** **of cases**
Total ADRs	142 (13.9)	205	57 (5.6)	86	6 (0.6)	7
**Neurological disorders**	117 (11.5)	146	49 (4.8)	66	4 (0.4)	5
Akathisia	64 (6.3)	65	24 (2.4)	24	2 (0.2)	2
Tremor	43 (4.2)	45	12 (1.2)	12	0 (0)	0
Dystonia	13 (1.3)	13	12 (1.2)	13	1 (0.1)	1
Parkinsonism	12 (1.2)	13	8 (0.8)	8	1 (0.1)	1
**Investigations**	26 (2.6)	27	7 (0.7)	9	2 (0.2)	2
Abnormal liver function	8 (0.8)	8	1 (0.1)	1	0 (0)	0
Weight gain	10 (1.0)	10	3 (0.3)	3	0 (0)	0
Blood prolactin increased	6 (0.6)	6	1 (0.1)	1	1 (0.1)	1
Blood glucose increased	2 (0.2)	2	0 (0)	0	1 (0.1)	1
White blood cell count decreased	0 (0)	0	1 (0.1)	1	0 (0)	0
Electrocardiogram QT prolonged, *n* (%)	1 (0.1)	1	0 (0)	0	0 (0)	0
**Gastrointestinal disorders**	5 (0.5)	6	3 (0.3)	3	0 (0)	0
**Eye disorders**	4 (0.4)	6	1 (0.1)	1	0 (0)	0
**Psychiatric disorders**	1 (0.1)	1	2 (0.2)	2	0 (0)	0
**Kidney and urinary disorders**	1 (0.1)	1	0 (0)	0	0 (0)	0

*ADRs, Adverse drug reactions*.

#### Subgroup Analysis of Akathisia and Extrapyramidal Symptoms

In different sex subgroups, the incidence of EPS ADRs was 12.8% in male patients and 19.0% in female patients. The incidence of akathisia ADRs was 7.1% in males and 10.0% in females (Supplementary Table S1). In different age subgroups, the incidence of ADRs to EPS was 29.5% in patients below 18 years old, 17.0% in patients aged 18–40 years old, and 11.7% in patients over 40 years old. The incidence of ADRs of akathisia was 19.2% in patients younger than 18 years, 8.5% in patients aged 18–40 years, and 6.6% in patients older than 40 years (Supplementary Table S2).

#### Subgroup Analysis of Weight Gain

The average weight of the patients before treatment and 12 weeks from baseline were 65.8 ± 14.16 kg, and 66.4 ± 13.95 kg, respectively. There was statistical significance in intra-group comparison before and after treatment (*P* < 0.05). 4.0% of patients had a ≥7% increase in body weight at 12 weeks from baseline (Supplementary Table S3).

In different sex subgroups, the mean weight change of male patients was 0.5 ± 2.81 kg while female patients was 0.3 ± 2.47 kg at 12 weeks after treatment. After 12 weeks of treatment, the weight increase of ≥7% in men from baseline was 4.4 and 3.7% in women (Supplementary Table S3).

In different age subgroups, the mean weight change was 0.2 ± 2.39 kg in patients below 18 years old, 0.4 ± 2.78 kg in patients aged between 18 and 40 years old, 0.3 ± 2.19 kg in patients older than 40 years old. The prevalence rate of weight gain ≥ 7% from the baseline was 8.1% in patients younger than 18 years old, 4.3% in patients aged 18–40 years old, and 2.2% in patients older than 40 years (Supplementary Table S4).

#### The Concomitant Medication

During the treatment, BNS concomitant medications accounted for 65.4%; concomitant EPS therapeutics or prophylactic medications 20.2%; concomitant EPS therapeutics 19.2%, and concomitant EPS prophylactic medications 1.3% (Supplementary Table S5).

### Effectiveness of BNS

In the effectiveness analysis population (1,018 patients), there were statistically significant differences in the group at each visit between post-treatment and pre-treatment (*P* < 0.001). Using LOCF method, the changes in total BPRS scores were −11.2 ± 10.17 (*N* = 1,018), −16.8 ± 12.69 (*N* = 1,018) and −20.6 ± 13.99 (*N* = 1,018) after 2/4, 6/8, or 12 weeks, respectively (Table 3). 53.5% (545/1,018) patients showed response to blonanserin treatment in week 12. And the changes in total BPRS scores without using LOCF method were −11.5 ± 10.13 (*N* = 990), −18.1 ± 12.52 (*N* = 900), and −22.8 ± 13.35 (*N* = 830) after 2/4, 6/8, or 12 weeks, respectively (Supplementary Table S6). 61.3% (509/830) patients showed response to blonanserin treatment in week 12. The 5-factor model scores were also significantly lower in patient after 2/4 weeks of treatment than that of baseline (*P* < 0.001), and continued to decrease thereafter (6/8 and 12 weeks after initiation of drug treatment). During surveillance, BPRS total score demonstrated the effectiveness of BNS clinical practice in patients with schizophrenia (Table 3).

**Table 3:** Mean changes of BPRS scores using LOCF method from baseline to 12 weeks after initiation of treatment.

**BPRS** **(mean ±SD)**	**Baseline** **(*N* = 1,018)**	**2/4 weeks** **(*N* = 1,018)**	**6/8 weeks** **(*N* = 1,018)**	**12 weeks** **(*N* = 1,018)**
**Total score**	48.7 ± 13.48	37.5 ± 11.03^**^	31.9 ± 9.89^**^	28.1 ± 9.51^**^
Change in total score	/	−11.2 ± 10.17	−16.8 ± 12.69	−20.6 ± 13.99
**Anxiety-depression**	9.9 ± 3.56	8.1 ± 2.89^**^	7.0 ± 2.61^**^	6.2 ± 2.39^**^
Change in anxiety-depression score	/	−1.8 ± 2.36	−2.9 ± 3.00	−3.6 ± 3.23
**Anergia**	10.0 ± 3.34	8.1 ± 2.75^**^	7.1 ± 2.45^**^	6.4 ± 2.34^**^
Change in anergia score	/	−1.9 ± 2.52	−2.9 ± 2.99	−3.6 ± 3.23
**Thought disturbance**	12.1 ± 4.25	9.2 ± 3.58^**^	7.6 ± 3.22^**^	6.6 ± 3.06^**^
Change in thought disturbance score	/	−2.9 ± 3.03	−4.4 ± 3.74	−5.5 ± 4.17
**Activation**	6.7 ± 2.98	5.1 ± 2.19^**^	4.4 ± 1.82^**^	4.0 ± 1.60^**^
Change in activation score	/	−1.6 ± 2.23	−2.3 ± 2.68	−2.7 ± 2.85
**Hostility-suspiciousness**	10.1 ± 3.94	7.1 ± 2.94^**^	5.8 ± 2.51^**^	4.9 ± 2.27^**^
Change in hostility-suspiciousness score	/	−3.0 ± 3.15	−4.3 ± 3.72	−5.1 ± 4.07

** *p < 0.001*.

*BPRS, Brief Psychiatric Rating Scale*.

## Discussion

### Safety

It is the first post-marketing surveillance of BNS in a Chinese population, including the differences in safety and effectiveness among different sexes and ages after drug administration. According to the PMS results, most AEs/ADRs are mild after drug taking, the incidence of severe AEs/ADRs is relatively lower.

The most common ADRs were akathisia, tremor, dystonia, and Parkinsonism, etc. According to data stratified by sex and age, female patients experienced a higher incidence of EPS ADRs than male patients do. The incidence of akathisia ADRs was also slightly higher in female patients. Younger patients experienced a higher incidence of EPS and akathisia ADRs than older ones do. There is no difference in weight changes from baseline was observed between patients with different sexes or ages.

#### Akathisia and Extrapyramidal Symptoms

The results of PMS showed that the most common ADRs in the Chinese population were akathisia, tremor, dystonia, Parkinsonism, etc. The incidence of EPS ADRs was 16.6%, which was lower than 48.46% of incidence rate in the Chinese population in phase 3 clinical trials (10). It is speculated that the inconsistency attributed to two reasons. First, in the phase 3 clinical trial, a smaller sample size and stricter monitoring requirements are needed to report every ADR that occurs in patients. The higher incidence in results is ascribed to the rigorous reporting. Secondly, the dosage of drug used in PMS study is fluctuating, that is, the adjustment of dosage was implemented based on the patient's tolerance. However, in the phase 3 clinical trial, a specified dose is added to the drug within a predetermined time. It is likely to make the patient's tolerance even worser. which is another possible cause for higher incidence in results. Nevertheless, the incidence rate is still slightly higher than that of Japanese PMS results. In other words, it indicated that the side effects of BNS may be ethnic-specific. A review article summarizing 5 types of PMS in Japan showed that the incidence of akathisia among patients subjected 12 week surveillance was 4.3% (14) while the incidence of akathisia in this study was 8.8%. In a 12-week PMS conducted in Japan, about 55% of patients had an illness duration with 10 years or more, whereas in Chinese PMS, the average duration of illness of patients was 5.3 years.

Generally, shorter duration of illness tends to show a higher sensitivity to antipsychotic drugs. The difference of illness duration in patients contributes to the discrepancy in the incidence of akathisia between Japanese and Chinese PMS. In addition, there is research to find another possible way to reduce EPS by converting blonanserin tablet/powder to transdermal patch. It is likely to attract our attention, deserves more research in the future. Overall, this study shows that relatively low incidence of akathisia and extrapyramidal side effects may be caused by BNS. Most of side effects are mild to moderate, and can be eliminated by using antagonists. It shows that the drug has good safety profile and tolerance. The analysis of ADRs stratified by sex showed that the incidence of EPS in male patients was 12.8%compared with 19.0% in female patients. The incidence of akathisia in male patients was 7.1%, while 10.0% in female patients. Women have slightly higher incidence of ADRs is than men. Sex differences in metabolism of antipsychotic drugs has been reported. The influence of sex on the metabolism of antipsychotic drugs has been reported a lot. Sex-related differences have been reported in a number of studies to discuss the relationships between sex and pharmacokinetics, gastric acid, intestinal motility, body weight and composition, blood volume, liver enzymes (mainly cytochrome P450) or renal excretion, etc. Plasma drug levels may be influenced by differences in sex-related parameters (15). Generally, women have lower gastric acid levels, resulting in slower gastrointestinal blood flow and longer gastric emptying time. Lipid cavities in women may make fat-soluble antipsychotic drugs accumulated easier and prolong the elimination half-life of the drug. In addition, the sex-related differences of drug metabolism reactions in the phase I (cytochrome P450) and phase II (such as glucuronidation) could also be one of the causes increasing side effects of antipsychotic drugs. For example, the lower activity of CYP1A2, CYP2C19, and CYP2E1 in women may lead to a decrease in drug clearance (16).

The results of analysis stratified by age showed that the incidence of ADRs in younger patients was slightly higher than that in older patients. Studies have found that some of the young people with mental illness who are medication-naïve tend to be more sensitive to drugs. Similarly, in five types of the PMS conducted in Japan, the incidence of ADRs in PMS was the highest 45.4%. This conclusion only reflects the treatment-naïve patients with first-episode schizophrenia (12). It means a higher dopamine occupation rate of the drug in younger patients could lead to a corresponding increase in EPS. It may be one of the possible reasons for a higher incidence of ADRs in younger patients. Another possible reason comes from the over-attention. The adolescent patients have captured more attention from family members and individuals, leading to a higher rate of reporting of ADRs.

#### Weight Gain

The results of the PMS of BNS showed that in Chinese patient group, only 4% of patients had weight gain more than 7% after taking BNS for 12 weeks. Its impact on weight gain is much less than other second-generation antipsychotic drugs. A national survey in Canada showed that after taking second-generation antipsychotics for 3 months, weight gain more than 7% of baseline was observed in 55.6% of patients taking quetiapine, 24.1% of patients taking olanzapine, and 23.7% of patients taking risperidone (17). The metabolic risk caused by antipsychotic drugs has been widely concerned by clinicians (18–20). Metabolic syndrome reduces the average life expectancy of patients with schizophrenia by 10–20 years, greatly affecting patient compliance (21, 22). The specific mechanism of weight gain and metabolic syndrome caused by antipsychotic drugs remains unclear. It may be related to decreased activity caused by genetic and drug-induced sedation, and increased appetite caused by receptors such as 5-HT2c and H1 (23–25). In the phase 3 clinical trial in China, BNS showed that the advantage of less weight gain is consistent with the PMS results in Japan.

This may be related to the lower 5-HT2c, H1, and M1 receptor affinity of BNS (4). It suggests that drugs like BNS with less impact on body weight and metabolism can be chosen for patients who have already had metabolic syndrome. In another study, BNS is recommended for use in diabetic patients, for it significantly reduce HbA1c and BMI compared with olanzapine. In this study, HbA1c data was not collected, and future research can make a in depth research in this direction (26). In analysis stratified by sex, there was no significant difference in weight gain between males and females. This is inconsistent with previous clinical reports. Previous studies have reported that weight gain is more common in female patients (27). However, no sex difference in weight found in the surveillance may be related to small increase in weight in the enrolled population. However, in analysis stratified by age, the result of younger patients gaining more weight is consistent with that of previous studies. Gentile found that age is one of the important factors affecting weight changes, and children and adolescents are risk factors for drug-induced weight gain (28, 29). Compared with adults, adolescent patients may gain more weight from the following two aspects. Firstly, it may be due to differences in microbial groups. It is generally believed that by the age of 3, our gut microbiota has been stable and consistent with that of adults. Yet, new research shows that gut microbiota maturation is a and continuous process with phylogenetic development differences. Even if it decreases with aging, there are still differences between adolescents and adults. The gut microbiota in adolescents are far less stable and diverse than that of adults. Therefore, the intestinal flora in adolescents tend to be more influenced by external factors than that in adults. This impact may last for a long time. long-term use of antipsychotic drugs may cause weight gain by affecting the stability of the intestinal flora (30). Secondly, due to hormonal changes during puberty, a significant increase in testosterone makes weight gain more prominent (31, 32). Furthermore, the overweight of adolescent patients may increase disease burden and suffer from stigma, as well as decrease social function, resulting in a decrease in medication compliance. Therefore, the weight gain induced by antipsychotic drugs, including BNS, may increase the risk of mental and physical health of adolescent patients. More attention should be paid to its use in adolescent patients.

#### Other Side Effects

Although BNS may cause some other minor side effects, such as liver damage, increased prolactin, etc., these influences can be alleviated by reducing the dose or using corresponding symptomatic treatment medicines. The increase of prolactin caused by antipsychotic drugs may cause irregular menstruation in women and sexual dysfunction in men, affecting patients' compliance. Risperidone has the most prominent effect on the increase of prolactin among the second-generation antipsychotic drugs. In early clinical trials, prolactin secretion less affected by BNS was confirmed by the results of this PMS in Chinese. Therefore, drugs like BNS with less effect on prolactin can be chosen for women of childbearing age for more cautious treatment.

### Effectiveness of BNS

Although the results in this study are unable to be directly compared with other large-scale, naturalistic, observational studies due to differences in drug class, race, and study duration, some similarities and differences were observed. In this study, before and after BNS treatment, the differences in BPRS scores within the group were statistically significant. With the extension of treatment time span, the BPRS score showed a gradual decline to indicate that the drug effectively improves the symptoms of schizophrenia patients, both in general population and subgroups of different sexes and ages. In previous phase 3 clinical trials in China and Japan, BNS have comparable efficacy with risperidone and haloperidol (4, 5). In a meta-analysis including 10 randomized controlled trials confirmed that risperidone was effective and better than aripiprazole (8). Post-marketing surveillance in Japan also reported the efficacy of BNS in long-term treatment (12). This surveillance once again proved the effectiveness of BNS in people with schizophrenia. However, the results in the rate of discontinue and response rate of BPRS are different from those in the past. In this study, patients with schizophrenia accepted treatment, 21.8% has dropped out of treatment by week 12 compared with 30% reported in a previous real-world study in Japan. In previous studies, the sample size was small with only 85 cases, and the duration illness was about 16.5 ± 11.7 years. It indicated that patients are likely to have a function decline, resulting to a high drug withdrawal rate. In addition, around 53.5% of patients experienced response to blonanserin treatment in week 12. Compared with other commonly used antipsychotic drugs like olanzapine, BNS experienced a lower response rate evaluated by BPRS. For example, a study pointed out that efficacy of olanzapine in BPRS was about 60%. Although olanzapine have a better response, the side effect of its long-term use on blood glucose and blood lipids cannot be ignored (33).

In conclusion, the post-marketing surveillance results of BNS show that the drug has good safety profile and effectiveness.

## Data Availability Statement

The original contributions presented in the study are included in the article/Supplementary Material, further inquiries can be directed to the corresponding author.

## Ethics Statement

The studies involving human participants were reviewed and approved by the Ethics Committee of leading site. Written informed consent to participate in this study was provided by the participants or their legal guardian/next of kin.

## Author Contributions

HW and JC contributed to the study concept, design, analysis, and interpretation. XW, XL, HS, QB, XY, ZX, KL, RZ, MS, DC, HD, GZhao, JL, XL, and GZhan contributed to data acquisition, data analysis, and interpretation. All authors reviewed and approved the final version of the manuscript. All authors contributed to the article and approved the submitted version.

## Conflict of Interest

The authors declare that this study received funding from Sumitomo Pharma Co., Ltd. and Sumitomo Pharma (Suzhou) Co., Ltd. The funder had the following involvement in the study: the study design and the analysis.

## Publisher's Note

All claims expressed in this article are solely those of the authors and do not necessarily represent those of their affiliated organizations, or those of the publisher, the editors and the reviewers. Any product that may be evaluated in this article, or claim that may be made by its manufacturer, is not guaranteed or endorsed by the publisher.
