# Supplementary material for: Safety and Effectiveness of Blonanserin in Chinese Patients with Schizophrenia: An Interim Analysis of a 12-Week Open-Label Prospective Multi-Center Post-marketing Surveillance
Source: Front Psychiatry. 2022 Aug 18;13:935769. doi: 10.3389/fpsyt.2022.935769 (PMC9435526; doi:10.3389/fpsyt.2022.935769)
Supplement: Supplementary file 1 [file Table_1.DOCX]

**Supplementary appendix**

Supplement to: Haishan Wu, Xijin Wang, Xuejun Liu, et al. Safety and effectiveness of blonanserin in Chinese patients with schizophrenia: an interim analysis of a 12-week open-label prospective multi-center post-marketing surveillance

**Appendix**

**Table of Contents:**

Appendix 1: Table S1 Analysis stratified by sex of akathisia and extrapyramidal symptoms

Appendix 2: Table S2 Analysis stratified by age of akathisia and extrapyramidal symptoms

Appendix 3: Table S3 Analysis stratified by sex of weight gain

Appendix 4: Table S4 Analysis stratified by age of weight gain

Appendix 5: Table S5 Proportion analysis of concomitant therapeutic and prophylactic agents with EPS

Appendix 6: Table S6 Mean changes of BPRS scores from baseline to 12 weeks after initiation of treatment

**Table S1** Analysis stratified by sex of akathisia and extrapyramidal symptoms

| ADRs | **Male** | **Female** |
| --- | --- | --- |
| **Total ADRs** n(%) | \| 66(16.6) \| \| --- \| | 139(22.4) |
| **EPS** n(%) | \| 51(12.8) \| \| --- \| | 118(19.0) |
| **Akathisia** n(%) | 28(7.1) | 62(10.0) |

ADRs= Adverse drug reactions; EPS=extrapyramidal symptoms

**Table S2** Analysis stratified by age of akathisia and extrapyramidal symptoms

|  | **<18 years** | **≥18 and ≤ 40 years** | **>40 years** |
| --- | --- | --- | --- |
| **Total ADRs** n(%) | 26(33.3) | 137(20.0) | 42(16.4) |
| **EPS** n(%) | \| 23(29.5) \| \| --- \| | 116(17.0) | 30(11.7) |
| **Akathisia** n(%) | 15(19.2) | \| 58(8.5) \| \| --- \| | 17(6.6) |

ADRs= Adverse drug reactions; EPS=extrapyramidal symptoms

**Table S3** Analysis stratified by sex of weight gain

| **Weight gain** | **Total** | **Female** | **Male** |
| --- | --- | --- | --- |
| Baseline (mean±SD) | 65.80±14.16 | 61.17±12.39 | 73.04±13.74 |
| 12 weeks (mean±SD) | 66.37±13.95^*^ | 61.55±11.96^*^ | 74.05±13.46^*^ |
| Change in body weight (mean±SD) | 0.36±2.61 | 0.28±2.47 | 0.48±2.81 |
| Weight increased from baseline≥7% n(%) | 33(4.0) | 19(3.7) | 14(4.4) |

* p＜0.05

**Table S4** Analysis stratified by age of weight gain

|  | **Total** | **<18 years** | | **≥18 and ≤ 40 years** | **>40 years** |
| --- | --- | --- | --- | --- | --- |
| Baseline (mean±SD) | 65.80±14.16 | | 58.08±14.15 | 66.99±14.66 | 64.95±11.88 |
| 12 weeks (mean±SD) | 66.37±13.95 | | 58.62±13.35 | 67.69±14.62^**^ | 65.34±11.54^*^ |
| Change in body weight (mean±SD) | 0.36±2.61 | | 0.24±2.39 | 0.39±2.78 | 0.31±2.19 |
| Weight gain from baseline≥7% n(%) | 33(4.0) | | 5(8.1) | 23(4.3) | 5(2.2) |

* p＜0.05, ** p<0.001

**Table S5** Proportion analysis of concomitant therapeutic and prophylactic agents with EPS

|  | **Used (n=1018)** |
| --- | --- |
| Total of proportion of concomitant medication for EPS | 206(20.2) |
| The proportion of prophylactic use of medication for EPS | 13(1.3) |
| The proportion of therapeutic use of medication for EPS | 195(19.2) |
| Proportion of medication for EPS use at the end of the study | 165(16.2) |

EPS=extrapyramidal symptoms

**Table S6** Mean changes of BPRS scores from baseline to 12 weeks after initiation of treatment.

| **BPRS (mean±SD)** | **Baseline**  **(N=1018)** | **2/4 weeks(N=990)** | **6/8 weeks(N=900)** | **12 weeks**  **(N=830)** |
| --- | --- | --- | --- | --- |
| **Total Score** | \| 48.7±13.48 \| \| --- \| | 37.3±10.88^**^ | \| 31.1±9.21^**^ \| \| --- \| | 26.2±7.68^**^ |
| change in total score | / | -11.5±10.13 | -18.1±12.52 | -22.8±13.35 |
| **Anxiety-depression** | 9.9±3.56 | 8.0±2.87^**^ | 6.9±2.51^**^ | 5.9±2.07^**^ |
| change in anxiety-depression score |  | -1.9±2.37 | -3.1±3.04 | -4.0±3.19 |
| **Anergia** | 10.0±3.34 | 8.1±2.73^**^ | 7.0±2.33^**^ | 6.1±2.06^**^ |
| change in anergia score | / | -1.9±2.53 | -3.1±3.02 | -4.0±3.20 |
| **Thought disturbance** | 12.1±4.25 | 9.1±3.53^**^ | 7.4±3.03^**^ | 6.0±2.47^**^ |
| change in thought disturbance score | / | -3.0±3.03 | -4.8±3.71 | -6.1±4.06 |
| **Activation** | 6.7±2.98 | 5.0±2.16^**^ | 4.2±1.70^**^ | 3.7±1.31^**^ |
| change in activation score | / | -1.7±2.25 | -2.5±2.74 | -3.0±2.89 |
| **Hostility-suspiciousness** | 10.1±3.94 | 7.0±2.90^**^ | 5.6±2.34^**^ | 4.5±1.79^**^ |
| change in hostility-suspiciousness score | / | -3.1±3.16 | -4.6±3.69 | -5.7±3.97 |

** p<0.001, BPRS= Brief Psychiatric Rating Scale
